# Application of Nanopore Sequencing for High Throughput Genotyping in Horses

**DOI:** 10.3390/ani13132227

**Published:** 2023-07-06

**Authors:** Artur Gurgul, Igor Jasielczuk, Tomasz Szmatoła, Sebastian Sawicki, Ewelina Semik-Gurgul, Bogusława Długosz, Monika Bugno-Poniewierska

**Affiliations:** 1 Center of Experimental and Innovative Medicine, University of Agriculture in Krakow, al. Mickiewicza 24/28, 30-059 Krakow, Poland; igor.jasielczuk@urk.edu.pl (I.J.); tomasz.szmatola@urk.edu.pl (T.S.); 2 Department of Animal Reproduction, Anatomy and Genomics, University of Agriculture in Krakow, al. Mickiewicza 24/28, 30-059 Krakow, Poland; sebastian.sawicki@student.urk.edu.pl (S.S.); boguslawa.dlugosz@urk.edu.pl (B.D.); monika.bugno-poniewierska@urk.edu.pl (M.B.-P.); 3 Department of Animal Molecular Biology, National Research Institute of Animal Production, Krakowska 1, 32-083 Balice, Poland; ewelina.semik@iz.edu.pl

**Keywords:** DNA variants, *Equus caballus*, genome enrichment, Oxford Nanopore, SNPs

## Abstract

**Simple Summary:**

Detection and genotyping of genetic variants across genomes has several applications that include, e.g., identification of genetic background of phenotypic traits, detection of disease-related variation, and analysis of population genetic structure. In this study, we attempt to develop a Nanopore sequencing-based genotyping strategy (with MinION system from Oxford Nanopore) that allows simple and cost-efficient genome-wide analysis in horse species. With this method, we generated 28,426 polymorphisms that were genotyped with high accuracy, with a level of error not exceeding 3%. The method can be further improved to increase the number of detected variants and improve their reliability by increasing the sequencing depth.

**Abstract:**

Nanopore sequencing is a third-generation biopolymer sequencing technique that relies on monitoring the changes in an electrical current that occur as nucleic acids are passed through a protein nanopore. Increasing quality of reads generated by nanopore sequencing systems encourages their application in genome-wide polymorphism detection and genotyping. In this study, we employed nanopore sequencing to identify genome-wide polymorphisms in the horse genome. To reduce the size and complexity of genome fragments for sequencing in a simple and cost-efficient manner, we amplified random DNA fragments using a modified DOP-PCR and sequenced the resulting products using the MinION system. After initial filtering, this generated 28,426 polymorphisms, which were validated at a 3% error rate. Upon further filtering for polymorphism and reproducibility, we identified 9495 SNPs that reflected the horse population structure. To conclude, the use of nanopore sequencing, in conjunction with a genome enrichment step, is a promising tool that can be practical in a variety of applications, including genotyping, population genomics, association studies, linkage mapping, and potentially genomic selection.

## 1. Introduction

Long-read sequencing technologies are the most recent innovations in genetic analysis. Earlier techniques, including next-generation sequencing (NGS) techniques such as sequencing by synthesis (e.g., Illumina sequencers, San Diego, CA, USA) and semiconductor sequencing (e.g., Ion Torrent instruments from Thermo Fisher Scientific) [1], are limited in the length of the molecules they are capable of sequencing thereby hampering the ability of the reads to be properly assembled. This limitation is especially problematic in repetitive genome regions in which assembly and mapping are particularly challenging [2]. Furthermore, NGS systems are exceptionally expensive and thus cost-prohibitive for small local laboratories. 

Third-generation sequencing platforms were developed to improve upon these limitations, focusing on maximizing the read length [3]. Two such techniques, associated with two different sequencing platforms, are currently available and widely applied: the more expensive PacBio system, utilizing HiFi sequencing technology, and the more affordable Oxford Nanopore, utilizing nanopore technology [3]. Nanopore sequencing involves monitoring the changes that occur in an electrical current as nucleic acids are passed through a protein nanopore. Nanopore sequencing devices use flow cells which contain an array of microscopic pores—nanopores—embedded in an electro-resistant membrane. Each nanopore is fitted with an electrode connected to a channel and sensor chip, which measures the electric current that flows through the nanopore. When a DNA or RNA molecule passes through a nanopore, the current is disrupted to produce a characteristic signal. The signal is then decoded using base calling algorithms to determine the DNA or RNA sequence in real-time (https://nanoporetech.com/ accessed on 20 June 2023). This method enables direct, high-throughput, real-time analysis of long DNA or RNA particles [4]. The increasing (together with chemistry, flow cells, and base calling algorithms development) quality of reads generated by nanopore sequencing systems (e.g., MinION, GridION, and PromethION, Oxford Nanopore) encourages their application in genome sequencing aimed at genome-wide polymorphism detection and genotyping. 

Genotyping-by-sequencing (GBS) is a specific NGS-based technique that allows for quick polymorphism detection and across-genome genotyping in large populations [5,6]. It involves reducing genome complexity by first digesting the DNA using carefully selected restriction enzymes (RE) and then size-selecting and sequencing the obtained fragments. The utility of this technique has been illustrated in several animal studies [6,7,8], and its effectiveness suggests that similar, though more optimized, approaches might be employed for third generation techniques, especially when affordable, personal systems such as MinION are available. The MinION system (especially the Mk1C version) is a small portable sequencing device that is able to produce (at perfect conditions) up to 50 Gb of sequence per flow cell in 72 h (https://nanoporetech.com/products/minion accessed on 20 June 2023). These systems can be purchased for a small fraction of the price of the Illumina systems and thus are available to any lab that would like to implement high-throughput genotyping. Furthermore, instead of the time-consuming optimization steps of RE digestion as in GBS [6], an alternative method of reducing genome complexity might be employed: degenerate oligonucleotide-primed polymerase chain reaction (DOP-PCR) [9]. Using degenerated primer (DOP-primer), this low-cost and simple method that requires only primers and PCR reagents employs few optimization steps and involves two stages of amplification, one at low and the second at high stringency. The resulting amplification products from most of the studied genomes are evenly scattered across the genome and range in size from about 150 to 1500 bp [9] and thus are at lengths suitable for nanopore sequencing techniques. DOP-PCR performance was tested on a variety of genomes [9], which makes DOP-PCR-based enrichment approaches potentially adaptable to genotyping in different species without major optimization steps. 

High-throughput, population-wide genotyping is of great interest in animal breeding and animal science. Not only can it be used in association and genetic diversity studies, quantitative trait loci mapping, selection signatures detection, and runs-of-homozygosity identification but also for genetic marker-based selection in animals [10,11,12,13,14]. Thus, developing a simple, inexpensive method of genome-wide genotyping of horse genomes could be beneficial to the horse breeding industry and scientists who study horse genomics.

The aim of this study was to develop a GBS-like approach to nanopore sequencing for use in variant detection and whole-genome genotyping in the horse. To this end, we performed genome complexity reduction using a modified DOP-PCR, and we sequenced the resulting amplified DNA fragments using a MinION Mk1C system. We then detected variants within those reads, validated them by Sanger sequencing, and evaluated their quality and reliability. 

## 2. Material and Methods

### 2.1. Material and DOP-PCR

The genomic DNA was extracted from peripheral blood or semen of 53 horses. Most of them (*n* = 50) were Huculs; the remaining 3 were Arabians (*n* = 1) or warmbloods (*n* = 2; part-bred Arabian and Trotter). The analyzed population included 37 females and 16 males of various ages, born between 2000 and 2021. All animal procedures were approved by the Local Ethics Committee in Krakow, resolution No. 98/2021, in accordance with the legal regulations of the European Union. Genomic DNA was purified using a Sherlock AX kit (A&A Biotechnology, Gdansk, Poland) and controlled for quality using agarose gel electrophoresis. A modified DOP-PCR [9] with standard primer (5′-CCCGACTCGAGNNNNNNATGTGG-3′) was applied to reduce genome complexity and enrich random genomic regions. The additional DOP-PCR modification introduced in this study involved application of a fast-PCR kit (QIAGEN Fast Cycling PCR Kit) to shorten the amplification procedure. The PCR mixture was as follows: 10 µL of Fast Cycling master mix, 1 µL of DOP-primer (65 µM), and 100–300 ng of genomic DNA supplemented with nuclease-free water up to 20 µL of total reaction volume. The cycling conditions were as follows: 5 min of initial denaturation at 95 °C, followed by 6 cycles of low stringency amplification and 38 cycles of high stringency amplification. The low stringency cycles included 5 s of denaturation at 96 °C, 10 s of annealing at 30 °C, a slow ramp at 0.3 °C/s to 68 °C, and 20 s of extension at 68 °C. The high stringency cycles included 5 s of denaturation at 96 °C, 10 s of annealing at 56 °C, and 20 s of extension at 68 °C. All cycles were followed by extending the final product for 1 min at 72 °C. The PCR products were analyzed on an agarose gel for size distribution, ensuring that there were no overamplified sequences, and they were then purified using AMPure XP Beads (Beckman Coulter, Brea, CA, USA) using a standard 1.8× protocol.

### 2.2. Nanopore Library Construction and Sequencing

The cleaned PCR products were quantified using a Qubit dsDNA kit (Thermo Fisher, Waltham, MA, USA) and were transformed into a sequencing library using a PCR Barcoding kit (Oxford Nanopore). This kit uses transposase which simultaneously cleaves template molecules in each sample and attaches tags, which contain primer binding sites, to the cleaved ends. Twelve primers were then used to amplify each sample: each primer contains a barcode and 5′ tag, which facilitates the ligase-free attachment of Rapid Sequencing Adapters. Amplified and barcoded samples were then pooled together, and Rapid Sequencing Adapters were added to the pooled mix. To obtain a high library yield sufficient for flow cell loading, the protocol was optimized for short fragments: the initial DNA input was doubled (200 ng per sample), and all steps involving DNA purification with magnetic beads were done in a reaction-to-beads ratio of 1:1, thereby avoiding a too-stringent size selection of short fragments. Then, each MinION flow cell (R9.4.1) was loaded with 12 pooled and indexed libraries, optimized to 200 fM of the library. The products were sequenced on an Mk1C system until the flow cell pores became inactive, the timing of which varied across different flow cells. Raw sequencing reads were deposited in the NCBI Sequence Read Archive (SRA) database under BioProject accession number PRJNA932161.

### 2.3. Data Analysis

Raw sequencing signals were demultiplexed and base called using high-accuracy Guppy software pipeline (MinION v6.1.7; Oxford Nanopore, Oxford, Great Brittan). Adapter sequences were removed using Porechop (v0.2.4; ILRI Research Computing, Nairobi, Kenya), and the reads were filtered for quality using Fastp software (v0.23.1) [15], which trimmed the ends (20 nts) of low-quality reads and excluded reads with base quality lower than Q12. Filtered reads were mapped against the EquCab3.0 horse genome assembly (https://www.ensembl.org/Equus_caballus/Info/Index accesed on 23 November 2022) using Minimap2 software (v2.1) [16].

Variants in the mapped sequences were called using FreeBayes software (v1.3.6) (which is a Bayesian genetic variant detector designed to find small polymorphisms) [17] with minor modifications for long reads (https://github.com/freebayes/freebayes accesed on 12 December 2022). The detected variants were initially filtered using VCFtools [18] to remove those with cumulative quality lower than 300 and across-sample coverage lower than 150 reads. The remaining variants were annotated using Variant Effect Predictor (VEP) on ENSEMBL database [19] and then underwent population-level filtering, removing variants with more than 20% of missing observations, with minor allele frequency lower than 0.01 and with deviations from Hardy–Weinberg equilibrium at *p* < 1.0 × 10^−5^. Finally, multiallelic variants and indels were removed to create marker set more compatible with SNP-dedicated software.

### 2.4. Population Genetics

For the population genetics analysis, individuals with a high proportion of missing genotypes (missing > 20%, *n* = 17) were removed. The remaining individuals were used to analyze population differentiation and calculate standard population parameters (heterozygosity, inbreeding coefficient [F_IS_], effective population size [N_e_]); a principal components analysis was employed for visualization purposes. Most calculations were performed using PLINK software [20], though NeEstimator v2.1 was used to estimate effective population size [21], and STRUCTURE software [22] was used to analyze population structure. STRUCTURE was run 10 times per subpopulation (K, from K1 to K3), with 100,000 iterations and a 100,000 burn-in period each. The best K was inferred using StructureSelector software [23] using the method proposed by Evanno et al. [24]. Finally, CLUMPK software was used to visualize the results [25].

### 2.5. SNPs Validation

Fourteen variants were selected for validation using Sanger sequencing method in at least 5 horses used in discovery analysis, representing different genotypes; the variants fell into each quartile of quality score distribution. PCR primers were designed from the variants’ flanking sequences using Primer3 software [26] (Supplementary File S1), and PCR amplification was performed using QIAGEN Fast Cycling PCR Kit, with minor modifications to the number of PCR cycles applied for each fragment (Supplementary File S1). The PCR products were purified using ExoSAP-IT™ PCR Product Cleanup Reagent (Thermo Fisher, Waltham, MA, USA) and were sequenced under standard conditions using BigDye™ Terminator v3.1 Cycle Sequencing Kit (Applied Biosystems, Waltham, MA, USA); the unused dye terminators were removed using BigDye Xterminator™ Purification Kit (Applied Biosystems). Sequences were read using 3500 Genetic Analyzer (Thermo Fisher Scientific), and the chromatograms were analyzed for sequence variants using Chromas Lite software v2.6.6 (Technelysium Pty Ltd., South Brisbane, Australia).

## 3. Results

### 3.1. Sequencing and Variants Discovery 

**Figure 1:**
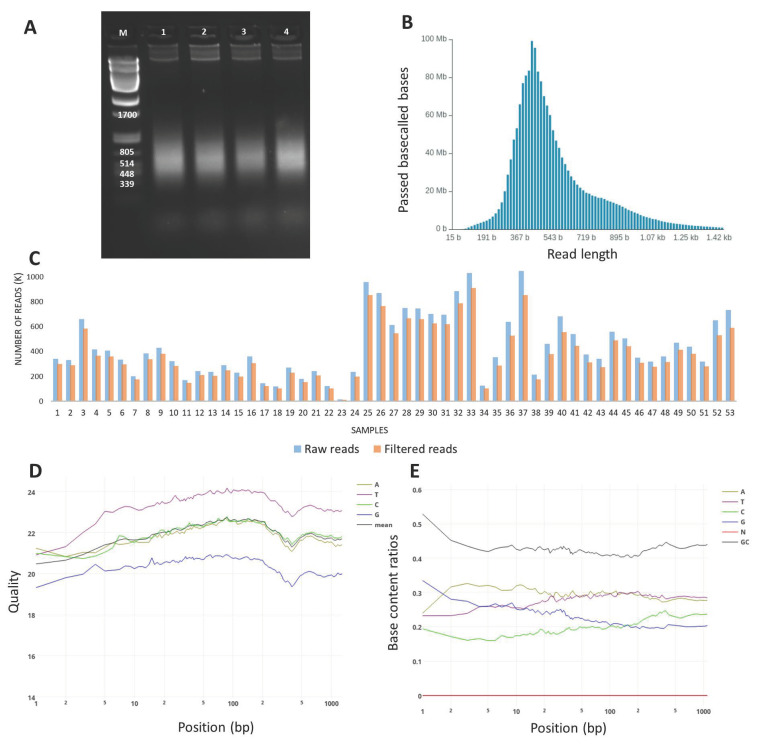
DNA fragments, reads, and sequencing statistics. (**A**)—Electrophoresis of DOP-PCR products in agarose gel. M-marker ladder; 1–4 samples. Clearly visible DNA fragments are generated in a size range of 200 to 1000 bp. (**B**)—Exemplary read length distribution for random sample. The read length distribution perfectly reflects the size of the obtained DOP-PCR products. (**C**)—Number of reads generated per sample (raw and filtered). Differences between samples are visible and are the result of differential sequencing output of separate flow cells. (**D**)—Mean reads quality for a randomly selected sample expressed in Phred quality score (Q). Most of the bases within the reads were of quality higher than Q15–Q20. (**E**)—Base content ratios within reads. Minor imbalance was observed in base content with slightly higher proportion of G base.

**Figure 2:**
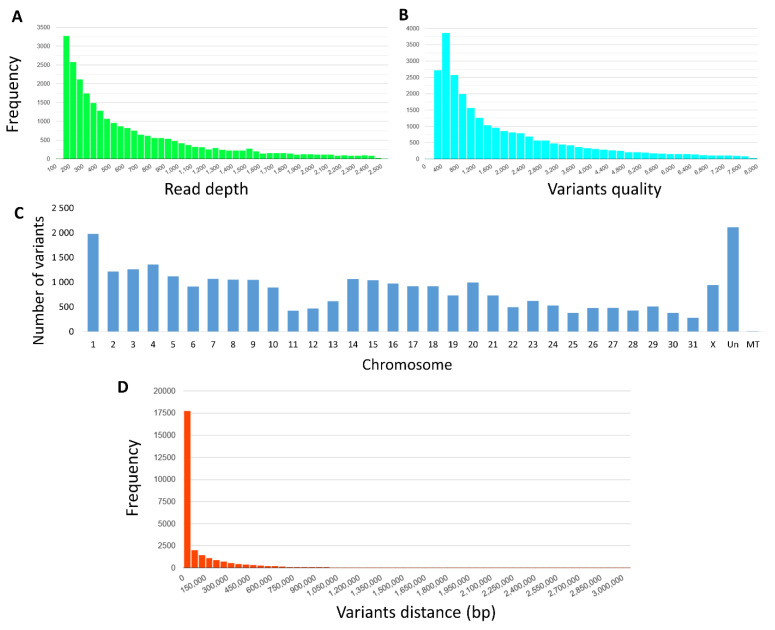
Detected variant statistics (initially filtered). (**A**)—Histogram of read depth at variants. Most of the variants were covered by up to 1000 reads, but some highly covered variants were also observed. (**B**)—SNPs quality distribution. (**C**)—Distribution of variants on chromosomes. (**D**)—Distribution of distances among nearest variants. Low proportion of large gaps between variants was observed. Un—unmapped contigs. MT—mitochondrion.

**Figure 3:**
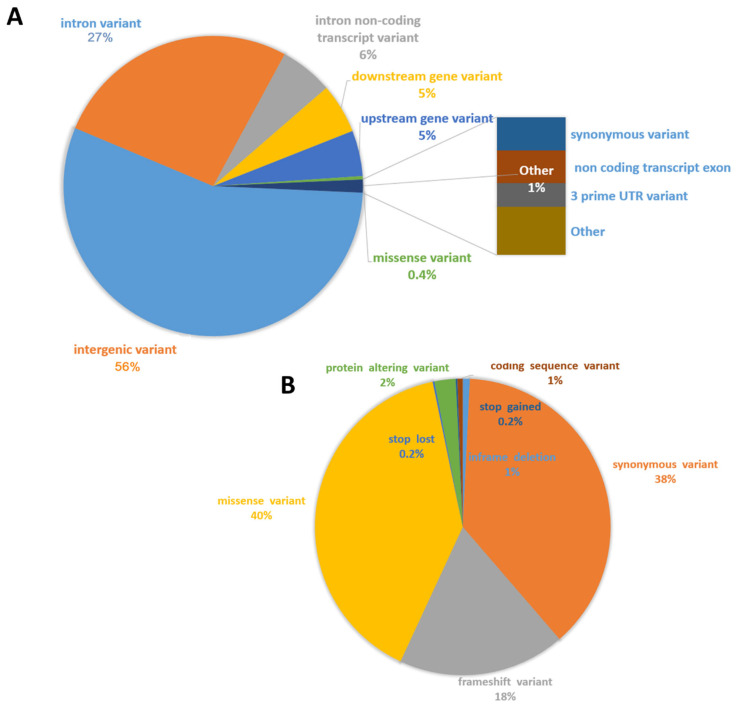
Annotation of the initially filtered variants. (**A**)—All variant consequences; (**B**)—Coding consequences.

The resulting DOP-PCR products ranged in size from 450–500 bp, as estimated on the agarose gel (Figure 1), and they were successfully transformed into sequencing libraries and sequenced on 5 R9 flow cells (around 12 samples per run). Depending on sequencing efficiency, we generated between 118.2 and 1046.5 K raw reads per sample (*x* = 452.0 K, SD = 241.4 K), except for one outlier with unusually poor performance, presumably the result of an accident (15.1 K reads). On average, 86% of reads passed filtering, and the average number of base pairs sequenced per sample was 161.2 Mbp (SD = 89.9) (Supplementary File S2). Of the sequenced bases, 64.8% had a quality score higher than Q20, and 14.2% had a score higher than Q30 (Figure 1). The filtered reads ranged in length from 337 to 499 bp and averaged 408 bp per sample (SD = 41.5) (Supplementary File S2). Of the filtered reads, 93.9%were successfully mapped against the most up-to-date horse genome (Supplementary File S3). Of the initial 167,052 variants detected, only 28,426 remained after filtering for coverage and quality. The resulting variants were found to be distributed on all horse chromosomes, with the highest concentration on ECA1 (*n* = 1975) and the lowest on ECA31 (*n* = 280) (821 on average per chromosome) (Supplementary File S4). Generally, the distribution of variants was correlated with the chromosome length (with Pearson correlation coefficient of 0.968) with the higher amount of variants on larger chromosomes. In unmapped contigs, 2115 variants were found, and 10 were found in mitochondrial DNA. The average distance between markers on chromosomes was 90.9 kb (SD = 190.6 kb) (Figure 2). For all variants across samples, the average quality score was Q4646 (range: Q300 to Q587,972; SD = 16,557), and the average coverage was 1277 (range: 151 to 102,810 reads; SD = 2978) (Figure 2). Variant annotation revealed 10,734 (37.8%) new variants previously not included in the ENSEMBL database. The variants overlapped with 4564 genes; an unexpectedly high proportion of variants was found in gene introns (27%), and another 56% of variants were classified as intergenic. About 0.9% (*n* = 520) were located in coding sequences, and 316 of them altered protein sequences (Figure 3). 

### 3.2. Application of Variants for Population Genetics

Only the variants of the highest quality were used as genetic markers in the population genetics analysis. Population-level filtering removed 11,358 variants due to an unacceptable number of missing genotypes, 427 due to the low minor allele frequency, and 299 due to departure from Hardy–Weinberg equilibrium. After removing variants other than SNPs, we retained 9495 high-quality genetic markers. Filtering for call rate across individuals removed another 17 horses from the final population (*n* = 36), leaving only Hucul breed individuals. For the remaining horses, the average amount of missing genotypes was 4.3% (SD = 4.1), and the minor allele frequency was 0.208 (SD = 0.135). See Supplementary File S5 for a distribution of these parameters across horses and markers. 

**Figure 4:**
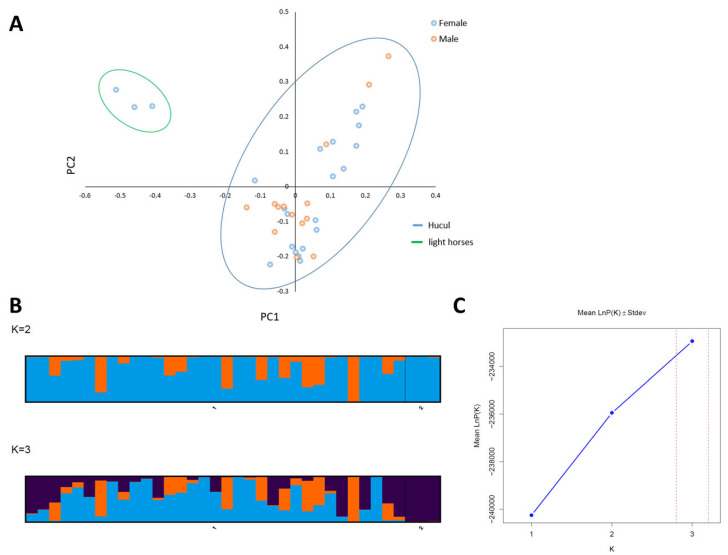
The visualization of genetic differentiation of all studied horses. (**A**)—PCA for the studied horses; (**B**)—Visualization of STRUCTURE results for K = 2 and K = 3; (**C**)—Result of Evanno analysis for the best K detection in STRUCTURE results. Red dotted line marks optimal K for the analyzed population.

The average inbreeding coefficient was slightly negative (F_is_ = −0.059, SD = 0.065), with an average observed heterozygosity of 0.304 (SD = 0.182) and average expected heterozygosity of 0.290 (SD = 0.150) (Supplementary File S6). The effective population size (N_e_) of Hucul horses was estimated to be 38.7 (linkage-disequilibrium method). Using all of the studied horses included in our study, the PCA visualization of the genetic differentiation indicated that the selected markers were able to properly differentiate the horse breeds (Hucul from non-Hucul), and it revealed some existing genetic variation within the Hucul breed (Figure 4). These results were confirmed with STRUCTURE; the Evanno method subdivided the population into three most-probable subpopulations (K = 3), whereas the simple delta K method subdivided it into two (K = 2) (Figure 4). The results confirmed the presence of internal genetic differentiation in the Hucul breed and provided support for the premise that the applied panel of markers has sufficient power to detect the genetic structure of horse breeds.

### 3.3. Variants Detection and Genotyping Accuracy—Validation Study

The results of the SNP validation are presented in Supplementary File S1. A total of 89 genotypes in 14 SNP loci were included in the validation. The presence of sequence variants at a *locus* was confirmed with 100% accuracy. Only three errors were detected in the assignment of genotypes, resulting in an overall genotyping error rate of 3.37%. Each of the errors occurred in different SNP loci, and they involved both homozygotic and heterozygotic genotypes in different samples. Also, unmatched reference alleles were observed in two SNP loci; this is likely the result of differences in the reference and analysis genomes. 

## 4. Discussion

Nanopore sequencing provides opportunities for the quick and inexpensive genome-wide analysis of multiple sample types [27]. Its high throughput allows for the identification of polymorphisms in large genomes, including mammalian genomes, and it has potential use in genotyping farm animals for genomic selection or pedigree control; this may be particularly useful when the systems necessary to perform these analyses are already on site (e.g., MinION from Oxford Nanopore) [28]. While this type of sequencing is known to produce low-quality reads, with errors up to 8% [29], this rate can be reduced to nearly 1% by increasing coverage and modifying the base calling algorithms [30]. 

Given the improvements that nanopore systems have made in data quantity and quality, we attempted to use enrichment-based nanopore sequencing to genotype a horse genome. As a model, we used the Hucul breed, which is a native mountain horse originating from the Carpathian Mountains. Using native horses for this analysis could presumably result in detecting more variants than would be possible in highly selected and inbred populations, but this statement needs further confirmation. We made several modifications to the standard sequencing procedures aimed at reducing the time and costs associated with the analysis and enabling genotyping at the population scale. First, we enriched random DNA fragments using the DOP-PCR technique, specifically employing fast-type reagents, to reduce the size and complexity of the to-be sequenced genome; to our knowledge, a similar approach has only been applied in plants, though using AFLP [31]. We also increased the amount of DNA we used for the library preparation process, modified size selection stringency, and the amount of library that had to be loaded to the flow cell.

While our methods were effective, there are areas for improvement. Unfortunately, we had low sequence output from the flow cells, and we assume that those with more experience working with Nanopore systems will be able to improve on our method and obtain better coverage of the enriched DNA fragments. Additionally, we were unable to use the system at full capacity, and we expect that doing so would improve the economics of the analysis, allow for a higher degree of multiplexing, and improve the quantity and quality of the identified variants.

Nevertheless, despite the low coverage, in this study, we identified 28,426 high-quality sequence variants. The number of variants detected was similar to that found in our previous study regarding GBS in horses using Illumina technology and low-depth sequencing [8]. A visibly higher number of variants was detected in single restriction enzyme-based enrichment approaches in, e.g., cattle 63,697 [7], but as we said before, the protocol presented here has the potential to be improved. 

Our results indicate that simply pre-filtering the variants obtained from nanopore sequencing improves the ability to identify high-quality polymorphisms. Furthermore, it does so at error rates of around 3% for detected genotypes, a rate satisfactory even for short-read NGS systems and lower than has been observed in a previous study using nanopore for SNP genotyping [32]. A similar error rate at a level of 3% was previously described for the Illumina-based GBS approach in cattle [33]. Such an error rate was also estimated in our previous study using short reads-based GBS in cattle, following a comparison with Illumina microarrays [8].

Likely due to the relatively low coverage (especially reproducibility in various samples, which were characterized by a different coverage), population-level filtering of the variants retained only 9495 SNPs for use as genetic markers. Despite this, the SNPs still seem to be capable of correctly describing the genetic structure of the studied horse breeds; not only were they capable of distinguishing between the breeds included in this study, but the estimated effective population size we obtained for Hucul horses (38.7) was very similar to sizes previously estimated using genotyping microarrays (40.3) [34] and other methods (37) [35], a finding that provides additional, if indirect, support for the usefulness of the proposed method. Indeed, nanopore sequencing has been proposed as a promising and versatile tool for SNP genotyping in humans for rapid and reliable perioperative risk prediction in clinical settings [36].

## 5. Conclusions

The results of this study suggest that nanopore sequencing is suitable for whole-genome genotyping of horse populations. This technique was able to generate short, high-quality sequence reads and identify their polymorphisms with satisfactory accuracy. Furthermore, the sequenced genotypes had high reliability and were of sufficient random distribution to be used as genomic markers in population genetics analyses. We suggest that, with the addition of minor methodological adjustments for optimization, the described protocol can be used for whole-genome genotyping in farm animals, thereby facilitating the implementation of genomic selection.

## Data Availability

Raw data were generated at University of Agriculture in Krakow. Derived data supporting the findings of this study are available from the corresponding author AG on request. Raw sequencing reads were deposited in the NCBI Sequence Read Archive (SRA) database under BioProject accession number PRJNA932161.

## Supplementary Materials

The following supporting information can be downloaded at: https://www.mdpi.com/article/10.3390/ani13132227/s1; Supplementary File S1: Primer sequences, SNPs, and PCR reaction conditions used in validation; Supplementary File S2: Number of raw and filtered reads generated per sample along with basic read statistics; Supplementary File S3: Read mapping statistics; Supplementary File S4: Basic statistics for the initially filtered sequence variants; Supplementary File S5: Minor allele frequency and missing genotypes distribution for population-based filtered variants; Supplementary File S6: Observed and expected heterozygosity per chromosome and average inbreeding (F_IS_) per sample based on population-based filtered variants.
