# Supplementary material for: Application of Nanopore Sequencing for High Throughput Genotyping in Horses
Source: Animals (Basel). 2023 Jul 6;13(13):2227. doi: 10.3390/ani13132227 (PMC10340048; doi:10.3390/ani13132227)
Supplement: Supplementary file 1 [file animals-13-02227-s001.zip › Supplementary File S2.pdf]

Table S1. Number of raw and filtered reads generated per sample along with basic read statistics.

Sample ID	Raw reads (K)	Filtered reads (K)	Total bases after filtering (M)	Q20 Bases (%)	Q30 bases (%)	Mean length after filtering (bp)
D1	340.1	301.1	117.8	66.8	15.8	391.0
D2	330.6	290.4	134.1	62.8	14.3	380.0
D3	661.2	585.2	218.3	62.9	13.7	373.0
D4	416.4	364.5	155.1	65.9	15.0	425.0
D5	405.9	358.5	146.7	62.7	13.4	409.0
D6	335.3	297.3	124.8	66.5	15.1	419.0
D7	201.8	177.5	70.4	66.2	15.1	396.0
D8	383.8	337.4	163.5	65.6	15.1	484.0
D9	430.5	382.3	162.2	66.1	14.8	424.0
D10	322.6	282.6	110.5	66.1	14.7	391.0
D11	169.8	149.4	66.8	66.7	14.3	446.0
D12	241.7	212.0	96.2	66.1	13.8	454.0
D13	237.3	205.9	69.5	65.7	16.4	337.0
D14	289.5	248.3	94.8	65.2	16.5	381.0
D15	230.9	199.0	72.1	65.4	15.9	362.0
D16	360.9	305.2	110.9	64.9	16.3	363.0
D17	144.7	123.6	48.1	64.4	14.8	389.0
D18	118.2	102.6	37.1	65.3	15.6	361.0
D19	269.9	230.4	88.3	64.7	15.6	383.0
D20	179.3	154.2	52.4	65.2	15.5	340.0
D21	242.7	208.3	86.5	65.0	15.9	415.0
D22	123.2	104.2	36.0	64.7	15.9	345.0
D23	15.1	12.6	4.5	65.2	15.9	358.0
D24	237.8	199.7	72.4	64.6	15.7	362.0
D25	956.8	851.2	302.2	66.3	13.2	355.0
D26	867.3	765.6	315.2	66.1	14.0	411.0
D27	613.4	546.9	212.5	66.4	13.6	388.0
D28	746.9	665.9	273.6	66.4	13.6	410.0
D29	743.8	660.5	272.1	66.3	13.9	411.0
D30	699.6	626.5	229.1	66.6	13.9	365.0
D31	694.8	620.3	294.0	66.2	13.8	473.0
D32	885.5	785.9	287.4	66.4	13.9	365.0
D33	1030.7	908.1	351.5	66.3	13.6	387.0
D45	126.8	104.0	42.1	63.0	13.3	405.0
D46	353.0	288.4	124.1	63.0	13.6	430.0
D47	638.1	526.0	235.6	63.2	13.2	447.0
D48	1046.5	852.3	423.6	62.9	13.7	497.0
D49	213.9	176.3	63.2	63.0	12.7	358.0
D50	459.3	377.5	166.7	62.9	13.0	441.0
D51	681.8	554.5	276.8	62.8	13.2	499.0
D52	538.3	443.6	208.4	62.9	13.0	469.0
D53	376.6	313.7	130.8	63.2	13.4	417.0
D54	340.4	275.9	133.5	62.7	13.0	483.0
D58	558.0	489.3	192.5	65.4	13.4	393.0
D59	505.6	441.4	175.7	65.3	13.6	398.0
D60	350.4	308.0	119.7	65.5	13.0	388.0
D61	318.4	276.5	116.7	65.0	13.5	422.0
D62	360.7	316.4	133.7	65.3	13.1	422.0

D81	471.2	413.7	173.8	65.3	13.1	420.0
D83	437.9	380.6	154.9	65.1	13.2	407.0
D84	319.9	281.9	111.7	65.4	13.4	396.0
D88	650.8	528.9	259.3	63.0	13.0	490.0
D89	732.6	591.0	268.5	62.4	12.6	454.0
All (mean)	449.9	388.3	161.2	64.8	14.2	408.3
