# Supplementary material for: Application of Nanopore Sequencing for High Throughput Genotyping in Horses
Source: Animals (Basel). 2023 Jul 6;13(13):2227. doi: 10.3390/ani13132227 (PMC10340048; doi:10.3390/ani13132227)
Supplement: Supplementary file 1 [file animals-13-02227-s001.zip › Supplementary File S4.pdf]

Table S2. Basic statistics for the initially filtered sequence variants.

Chromosome	Number of variants	Total Quality	Total depth at variant	Average distance (bp)
1	1,975	4,485	1,416	95,141
2	1,213	3,341	972	99,854
3	1,261	4,252	1,337	95,514
4	1,356	4,389	1,443	80,653
5	1,120	3,904	1,181	86,319
6	912	4,387	1,388	95,340
7	1,070	4,026	1,210	92,299
8	1,053	5,024	1,375	90,127
9	1,047	4,698	1,414	81,625
10	893	3,356	960	95,037
11	423	3,609	1,167	141,927
12	471	2,459	653	77,616
13	618	12,705	1,725	69,534
14	1,063	3,235	1,023	89,035
15	1,037	4,645	1,223	89,214
16	973	3,831	1,192	91,422
17	917	4,342	1,332	87,959
18	917	4,951	1,509	90,076
19	735	3,794	1,061	84,530
20	991	4,398	1,018	65,206
21	732	4,825	1,271	80,437
22	495	3,767	1,075	99,800
23	621	6,491	1,458	89,185
24	527	5,577	1,674	91,308
25	380	6,843	1,592	104,783
26	478	4,667	1,251	88,942
27	484	4,006	1,196	82,177
28	428	3,143	1,100	108,507
29	506	6,698	1,735	68,503
30	382	4,146	1,217	80,300
31	280	3,038	1,126	92,072
X	943	3,682	1,022	134,952
Contig	2,115	6,601	1,471	NA
MT	10	3,095	607	NA
All (sum/mean)	28,426	4,646	1,277	90,903
