# Supplementary figures and images for: Application of Nanopore Sequencing for High Throughput Genotyping in Horses
Source: Animals (Basel). 2023 Jul 6;13(13):2227. doi: 10.3390/ani13132227 (PMC10340048; doi:10.3390/ani13132227)

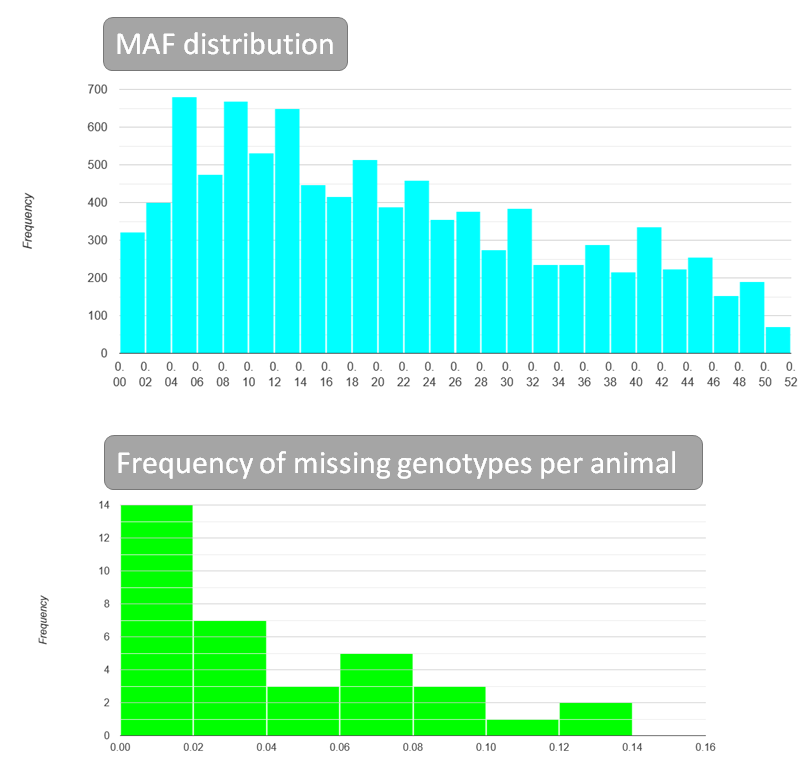

Supplement: Supplementary file 1 [file animals-13-02227-s001.zip › Supplementary File S5.png]

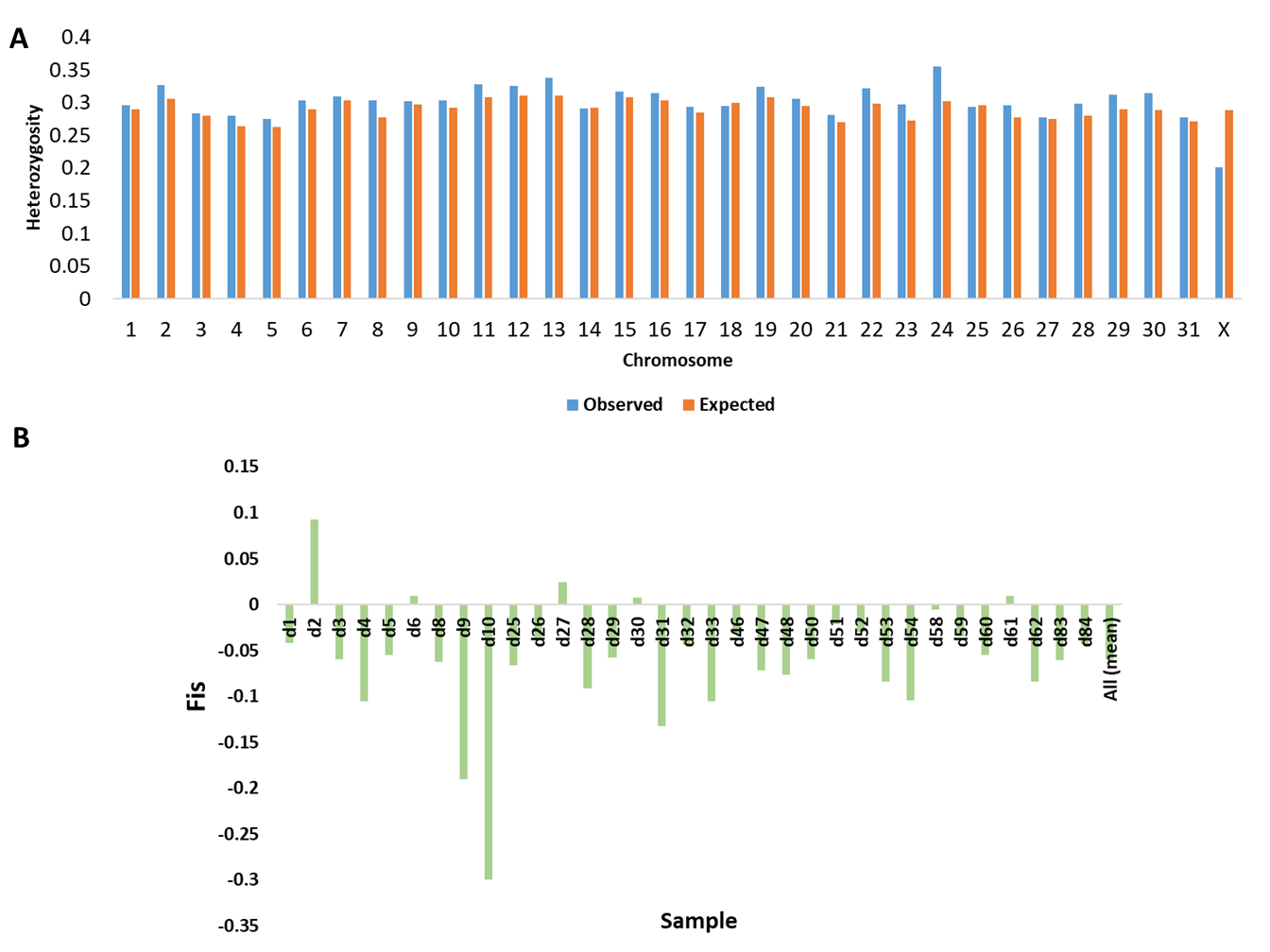

Supplement: Supplementary file 1 [file animals-13-02227-s001.zip › Supplementary File S6.png]
